# Supplementary material for: The impact of male circumcision on risk of HIV infection in sub-Saharan Africa: a household fixed-effects analysis of 279,351 men from 29 countries
Source: Front Public Health. 2026 Sep 29;14:1810573. doi: 10.3389/fpubh.2026.1810573 (PMC13658612; doi:10.3389/fpubh.2026.1810573)
Supplement: Supplementary file 1 [file Supplementary_file_1.pdf]

SUPPORTING INFORMATION

Supplementary Figure 1. Data availability by country and year. Surveys were included when self-reported male circumcision status and HIV blood-test result were available for males between 15 years of age and 49 years or older.

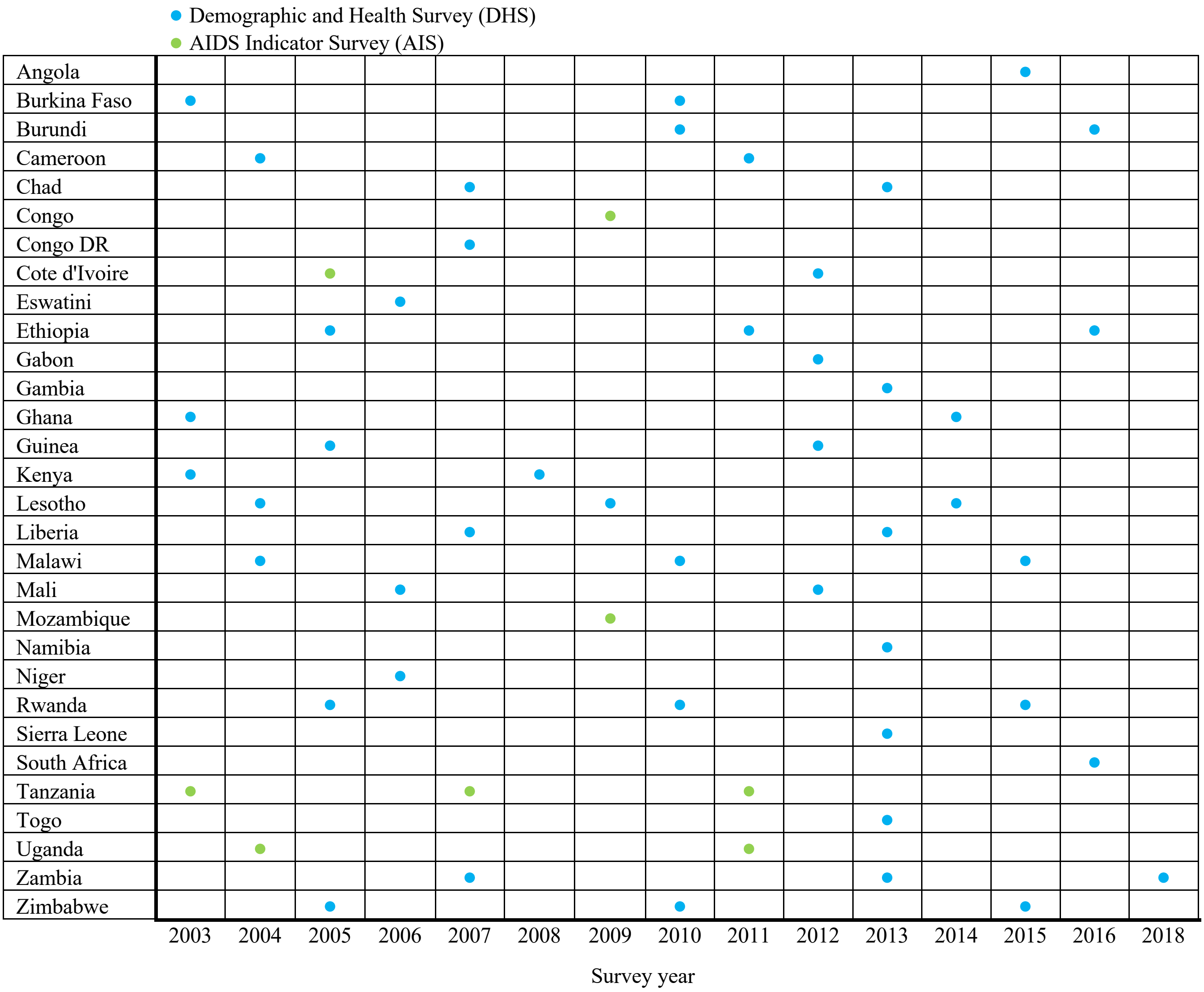

Supplementary Figure 2. Flow chart of the data selection.

	Sub-Saharan Africa, overall	Central Africa	Eastern Africa	Southern Africa	Western Africa
Available **Demographic and Health Surveys** (DHS) and **AIDS Indicator Surveys** (AIS) conducted in **sub-Saharan Africa** between **2000 and 2020**	**38** countries, **107** surveys	**7 countries, 14 surveys**	**13 countries, 44 surveys**	**4 countries, 8 surveys**	**14 countries, 41 surveys**
**Surveys** that include both **HIV and male circumcision indicators**	**31** countries, **61** surveys, **344,832** males	**5** countries, **7** surveys, **41,388** males	**5** countries, **12** surveys, **83,661** males	**9** countries, **18** surveys, **98,768** males	**12** countries, **24** surveys, **121,015** males
All men with known **HIV and circumcision status**	**279,351** males	**27,239** males	**63,687** males	**93,821** males	**94,604** males
**Two or more adult men per household**	**94,609** males	**10,600** males	**22,306** males	**33,208** males	**28,495** males
Two or more adult men per **household** where **at least one men is uncircumcised and one is circumcised** (discordant households)	**16,846** males	**2,763** males	**4,079** males	**7,963** males	**2,041** males

Supplementary Table 1. Start year of national voluntary medical male circumcision (VMMC) programmes in Eastern and Southern Africa – for the countries that were included in this study. Source: WHO and UNAIDS VMMC progress report (https://www.who.int/publications/i/item/voluntary-medical-male-circumcision-progress-brief-2019).

Country	Start year of national VMMC program
eSwatini	2008
Ethiopia	2009
Kenya	2008
Lesotho	2012
Malawi	2008
Mozambique	2009
Namibia	2009
Rwanda	2010
South Africa	2008
South Sudan	2017
Tanzania	2009
Uganda	2010
Zambia	2008
Zimbabwe	2009

Supplementary Figure 3. Maps show the African Regions and countries with national voluntary medical male circumcision (VMMC) programs.

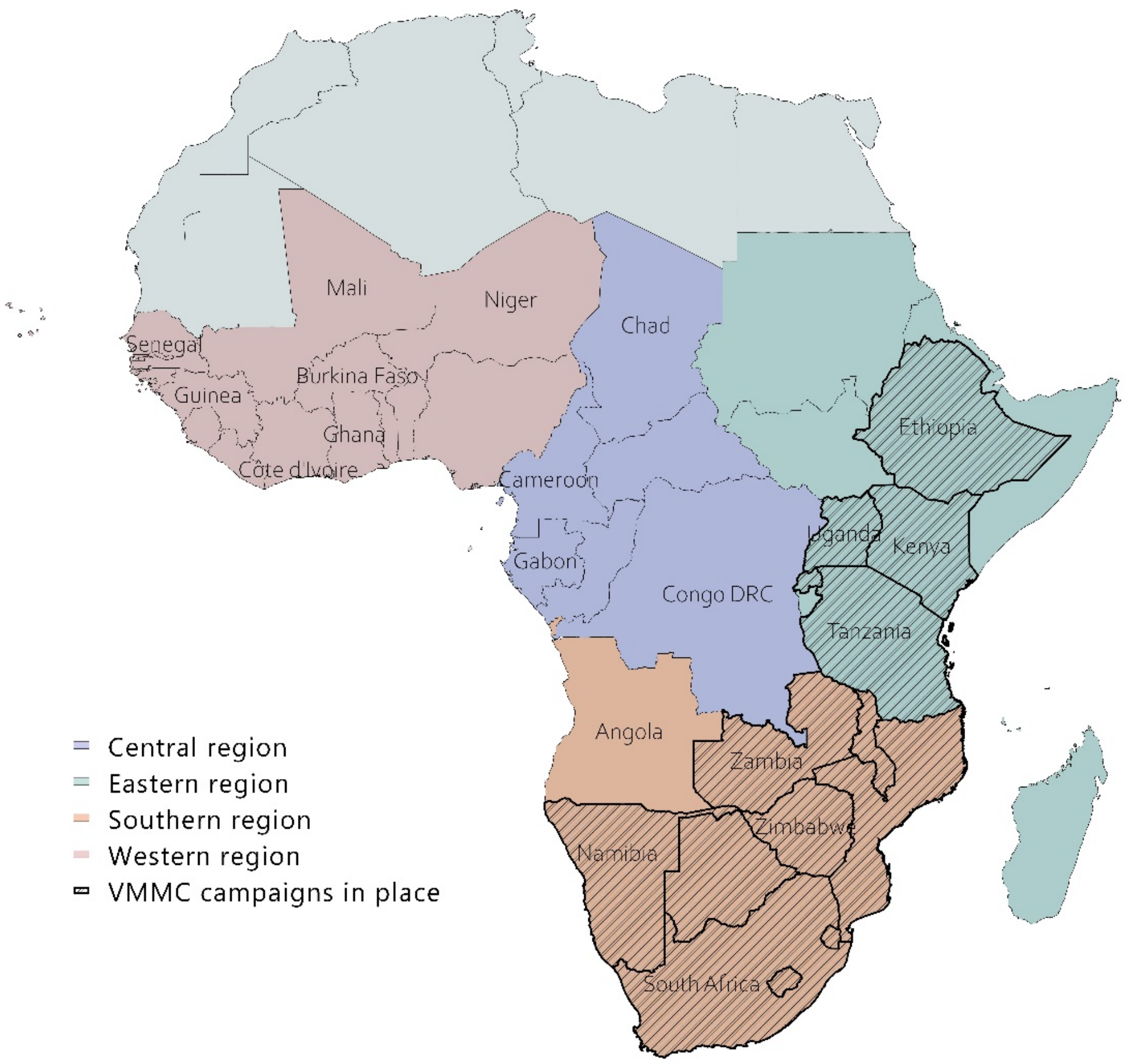

Supplementary Table 2. Modified Poisson regression models of the effect of male circumcision on HIV status among men in sub-Saharan Africa. Adjusted risk ratios (aRR) are shown with 95% confidence intervals (CIs), which are adjusted for heterogeneity at the household-level using fixed-effects. Schooling levels are defined as the highest level of education attended. Previously married includes being divorced, separated, or widowed. The number of observations (N) includes men from all households where at least two circumcision discordant men (*i.e.* at least one man circumcised and one uncircumcised) were included in the data. Residual degrees of freedom (DF) indicate the number of independent parameters estimated by the model. The pseudo R^2 represents the proportion of the total variability explained by the model.

	**Model 1 – nested model**		**Model 2 – adjusted for age**		**Model 3 – adjusted for age and educational level**		**Model 4 – adjusted for age, educational level and marital status**	
**Variable**	**aRR [95% CI]**	**p-value**	**aRR [95% CI]**	**p-value**	**aRR [95% CI]**	**p-value**	**aRR [95% CI]**	**p-value**
**Male circumcision status**								
Circumcised	0.83 [0.76–0.90]	<0.001	0.81 [0.74–0.88]	<0.001	0.81 [0.74–0.88]	<0.001	0.80 [0.73–0.88]	<0.001
Not circumcised	1 (ref)	-	1 (ref)	-	1 (ref)		1 (ref)	-
**Age**								
15-24			1 (ref)	-	1 (ref)	-	1 (ref)	-
25-34			3.32 [3.04–3.63]	<0.001	3.32 [3.04–3.64]	<0.001	2.57 [3.24–4.12]	<0.001
35-44			4.79 [4.39–5.23]	<0.001	4.81 [4.40–5.26]	<0.001	3.22 [5.13–6.60]	<0.001
45-54			5.19 [4.73–5.70]	<0.001	5.21 [4.74–5.73]	<0.001	3.42 [5.70–7.49]	<0.001
55+			3.68 [3.07–4.41]	<0.001	3.69 [3.07–4.42]	<0.001	2.29 [2.92–4.60]	<0.001
**Highest education**								
No education					1.04 [0.92–1.17]	0.541	1.06 [0.87–1.27]	0.612
Primary					1 (ref)	-	1 (ref)	-
Secondary					1.03 [0.95–1.12]	0.448	1.03 [0.96–1.18]	0.256
Post-secondary					0.98 [0.84–1.14]	0.796	0.98 [0.77–1.13]	0.476
**Marital status**								
Never married							1	-

Currently married				1.56 [1.40–1.72]	<0.001
Previously married				2.13 [1.88–2.40]	<0.001
**Model summary**					
N (observations)	19,911	19,911	19,910	18,836	19,910
Pseudo R^2	0.169	0.233	0.233	0.239	0.237

Supplementary Table 3. Modified Poisson regression model of the estimated effect of male circumcision on living with HIV (males aged 15 years or older) in sub-Saharan Africa, based on the sub-sample of men who live in the same household but differ in male circumcision status (discordant men).

	**Model – adjusted for age, educational level, and marital status**	
**Variable**	**aRR [95% CI]**	**p-value**
**Male circumcision status**		
Circumcised	0.80 [0.73–0.88]	<0.001
Not circumcised	1 (ref)	-
**Age**		
15-24	1 (ref)	-
25-34	2.34 [1.92–2.86]	<0.001
35-44	3.01 [2.39–3.79]	<0.001
45-54	3.19 [2.50–4.06]	<0.001
55+	2.01 [1.40–2.88]	<0.001
**Highest education**		
No education	1.05 [0.85–1.30]	0.651
Primary	1 (ref)	-
Secondary	0.96 [0.83–1.10]	0.536
Post-secondary	1.07 [0.82–1.40]	0.594
**Marital status**		
Never married	1	-
Currently married	1.53 [1.26–1.86]	<0.001
Previously married	1.86 [1.48–2.33]	<0.001
**Model summary**		
N (observations)	7,759	
Residual DF	6,669	
Pseudo R^2	0.277	

Supplementary Table 4. Outcomes for sub-group analyses. Adjusted risk ratios (aRR) are shown with 95% confidence intervals (CIs), which are adjusted for heterogeneity at the household-level using fixed-effects. The number of observations (N) includes men from all households where at least two circumcision discordant men (*i.e.* at least one man circumcised and one uncircumcised) were included in the data. Residual degrees of freedom (DF) indicate the number of independent parameters estimated by the model. The pseudo R^2 represents the proportion of the total variability explained by the model.

	**By African region**				**By age**		**By presence national VMMC programs**		**By family relatedness**
	**Central**	**Eastern**	**Southern**	**Western**	**34 or younger**	**35 or older**	**National VMMC programmes launched**	**No national VMMC programmes launched**	**Brothers**
**Model estimates**									
**Risk ratio**	0.88	0.63	0.81	0.84	0.79	0.82	0.75	0.98	0.78
95% CI	0.43–1.82	0.46–0.85	0.72–0.91	0.60–1.16	0.69–0.91	0.63–1.06	0.66–0.85	0.83–1.15	0.66–0.91
Robust SE	0.33	0.10	0.05	0.14	0.057	0.110	0.046	0.083	0.064
z-score	-0.35	-3.04	-3.60	-1.07	-3.28	-1.51	-4.69	-0.29	-3.07
p-value	0.727	0.002	<0.001	0.285	<0.001	0.130	<0.001	0.768	0.002
**Model summary**									
N (observations)	419	1,235	7,021	11,235	10,247	2,778	5,868	14,043	3,622
Residual df	251	729	4,065	10,663	8,590	2,415	3,410	12,329	2,111
Pseudo-R^2	0.168	0.159	0.158	0.170	0.208	0.176	0.151	0.230	0.100

Supplementary Table 5. Comparison of data characteristics for all men included in the surveys versus men from circumcision discordant households.

	All men N (%)	More than one man, discordant circumcision status N (%)
**Sample size**	344,832	18,182
**HIV status**		
Positive	15,952 (4.7%)	1,078 (6.0%)
Negative	322,794 (95.3%)	16,855 (94.0%)
**Male circumcision status**		
Circumcised	183,460 (64.7%)	9,184 (53.8%)
Not circumcised	100,088 (35.3%)	7,904 (46.3%)
**Sociodemographic characteristics**		
**Age (per 10-year age group)**		
34 or younger	224,050 (65.0%)	13,065 (71.9%)
35 and older	120,782 (35.0%)	5,117 (28.1%)
**Highest education**		
No education	75,403 (21.9%)	2,117 (11.6%)
Primary	129,201 (37.5%)	7,138 (39.3%)
Secondary	120,958 (35.1%)	7,673 (42.2%)
Post-secondary	19,245 (5.6%)	1,253 (6.9%)
**Socioeconomic status**		
1 'Poorest'	66,980 (19.4%)	2,737 (15.1%)
2	66,485 (19.3%)	2,935 (16.1%)
3	65,708 (19.1%)	3,159 (17.4%)
4	67,275 (19.5%)	3,714 (20.4%)
5 'Wealthiest'	78,381 (22.7%)	5,637 (31.0%)
**Marital status**		
Never married	141,046 (41.6%)	10,771 (59.2%)
Currently married	184,183 (54.3%)	6,639 (36.5%)
Previously married	13,941 (4.1%)	772 (4.3%)
**(Sexual) behavioural characteristics**		
**Lifetime sex partners**		
None	97,150 (29.2%)	5,964 (33.7%)
1-2	89,459 (26.9%)	4,166 (23.6%)
3-6	88,331 (26.5%)	4,373 (24.7%)
7+	58,127 (17.5%)	3,179 (18.0%)
**Had STI during past 12 months**		
Yes	18,541 (5.4%)	1,072 (5.9%)
No	325,962 (94.6%)	17,096 (94.1%)
**Ever engaged in transactional sex**		
Yes	19,794 (11.1%)	994 (12.3%)
No	159,146 (88.9%)	7,066 (87.7%)
**Survey characteristics**		
**Region**		
Central Africa	41,388 (12.0%)	3,689 (20.3%)
Eastern Africa	83,661 (24.3%)	4,147 (22.8%)
Southern Africa	98,768 (28.6%)	8,197 (45.1%)
Western Africa	121,015 (35.1%)	2,149 (11.8%)
**Time of survey**		
Between 2000-2004	40,308 (11.7%)	1,390 (7.6%)
Between 2005-2009	106,046 (30.8%)	5,537 (30.45%)
Between 2010-2014	136,072 (39.5%)	6,068 (33.4%)
Between 2015-2019	62,406 (18.1%)	5,187 (28.5%)

Supplementary Table 6. Comparison of data characteristics for circumcised men from single-men households, circumcised men from multiple-men households, uncircumcised men from single-men households, and uncircumcised men from multiple-men households.

	Circumcised men from single-men households N (%)	Circumcised men from multiple-men households N (%)	Uncircumcised men from single-men households N (%)	Uncircumcised men from multiple-men households N (%)
**Sample size**	122,532	59,164	65,038	36,814
**HIV status**				
Positive	116,614 (3.6%)	1,462 (2.5%)	6,366 (10.0%)	2,459 (6.8%)
Negative	4,410 (96.4%)	57,016 (97.5%)	57,352 (90.0%)	33,672 (93.2%)
**Sociodemographic characteristics**				
**Age (per 10-year age group)**				
34 or younger	73,801 (60.2%)	42,644 (72.1%)	40,943 (63.0%)	26,801 (72.8%)
35 and older	48,731 (39.8%)	16,520 (27.9%)	24,095 (37.0%)	10,013 (27.2%)
**Highest education**				
No education	36,723 (30.0%)	12,016 (20.3%)	7,720 (11.9%)	3,043 (8.3%)
Primary	39,270 (32.1%)	19,569 (33.1%)	31,515 (48.5%)	17,414 (47.3%)
Secondary	38,887 (31.7%)	23,656 (40.0%)	22,563 (34.7%)	14,704 (39.9%)
Post-secondary	7,647 (6.2%)	3,920 (6.6%)	3,235 (5.0%)	1,652 (4.5%)
**Socioeconomic status**				
1 'Poorest'	25,748 (21.0%)	8,982 (15.2%)	13,919 (21.4%)	5,453 (14.8%)
2	24,519 (20.0%)	9,764 (16.5%)	13,444 (20.7%)	6,462 (17.6%)
3	22,748 (18.6%)	10,583 (17.9%)	13,035 (20.4%)	7,636 (20.7%)
4	22,084 (18.0%)	12,239 (20.7%)	13,008 (20.0%)	8,179 (22.2%)
5 'Wealthiest'	27,432 (22.4%)	17,596 (29.4%)	11,632 (17.9%)	9,082 (24.7%)
**Marital status**				
Never married	78,995 (64.5%)	21,469 (36.3%)	41,757 (64.2%)	12,172 (34.5%)
Currently married	38,031 (31.0%)	35,359 (59.8%)	20,407 (31.4%)	23,344 (63.4%)
Previously married	5,506 (4.5%)	2,335 (4.0%)	2,872 (4.4%)	1,298 (3.5%)
**(Sexual) behavioural characteristics**				
**Lifetime sex partners**				
None	27,562 (23.7%)	23,156 (40.7%)	12,916 (20.3%)	13,742 (38.0%)
1-2	31,506 (27.0%)	12,375 (21.7%)	20,840 (32.8%)	9,505 (26.3%)
3-6	32,508 (27.9%)	12,331 (21.7%)	20,417 (32.1%)	8,898 (24.6%)
7+	24,988 (21.4%)	9,089 (16.0%)	9,443 (14.8%)	4,056 (11.2%)
**Had STI during past 12 months**				
Yes	6,969 (5.7%)	2,893 (4.9%)	4,033 (6.2%)	1,801 (4.9%)
No	115,472 (94.3%)	56,233 (95.1%)	60,951 (93.8%)	34,976 (95.1%)
**Ever engaged in transactional sex**				
Yes	6,497 (9.9%)	2,895 (10.2%)	4,558 (12.2%)	2,028 (12.4%)
No	59,264 (90.1%)	25,640 (89.9%)	32,912 (87.8%)	14,280 (87.6%)
**Survey characteristics**				
**Region**				
Central Africa	12,192 (10.0%)	7,933 (13.4%)	4,459 (6.9%)	2,683 (7.3%)
Eastern Africa	25,896 (21.1%)	13,351 (22.6%)	15,943 (24.5%)	9,194 (25.0%)
Southern Africa	20,340 (16.6%)	10,423 (17.6%)	41,855 (64.4%)	23,555 (64.0%)
Western Africa	64,104 (52.3%)	27,457 (46.4%)	2,781 (4.3%)	1,382 (3.8%)
**Time of survey**				
Between 2000-2004	18,891 (15.4%)	9,962 (16.8%)	2,416 (3.7%)	1,499 (4.1%)
Between 2005-2009	50,256 (41.0%)	19,231 (32.5%)	15,420 (23.7%)	8,449 (23.0%)
Between 2010-2014	38,568 (31.5%)	21,790 (36.3%)	28,368 (43.6%)	16,578 (45.0%)
Between 2015-2019	14,817 (12.1%)	8,181 (13.8%)	18,834 (29.0%)	10,288 (28.0%)
